# Posterior capsule dynamics during femtosecond laser lens fragmentation

**DOI:** 10.1007/s10792-023-02739-6

**Published:** 2023-05-16

**Authors:** Carlo Bellucci, Paolo Mora, Salvatore Antonio Tedesco, Roberto Bellucci, Stefano Gandolfi

**Affiliations:** 1 Ophthalmology Unit, Department of Medicine and Surgery, University of Parma, Via Gramsci 14, 43126 Parma, Italy; 2 Vista Vision Surgical Centre, Verona, Italy

**Keywords:** Cataract surgery, Femtosecond laser, Posterior capsule rupture, Posterior capsule dynamics

## Abstract

**Purpose:**

The dynamics of the posterior capsule during femtosecond laser lens fragmentation has received little attention in the literature. We analysed the movements of the posterior capsule to identify the rupture risk factors, if any, and to suggest possible modification of the laser spot energy pattern during fragmentation.

**Materials and methods:**

Posterior capsule ruptures during fragmentation were identified over a 10-year period of femtosecond laser use. In addition, the dynamics of the posterior capsule were identified through the real-time swept-source OCT lateral view available during the surgeries.

**Results:**

Out of the 1465 laser cataract procedures performed, we recorded 1 case of posterior capsule rupture during lens fragmentation, which was caused by eye movement that was detected but ignored by the surgeon. Three types of posterior capsule dynamics were identified, all related to a gas bubble formation during the first part of the lens fragmentation. In eyes with a hard nucleus, the concussion of the posterior capsule was evident, however, with no capsule rupture.

**Discussion:**

Maintaining good docking throughout the whole procedure seems important in avoiding a posterior capsule cut by the femtosecond laser. In addition, a Gaussian pattern of spot energy is suggested when fragmenting hard cataracts.

**Supplementary Information:**

The online version contains supplementary material available at 10.1007/s10792-023-02739-6.

## Introduction

Femtosecond lasers have been used for cataract surgery since 2009 [1]. Supporters of this technology point out the precision of the capsulotomy, the efficacy of fragmentation and the predictability and the sealing properties of incisions [2]. Despite these advantages, the femtosecond laser-assisted cataract surgery (FLACS) has never become popular. The cost of the procedure, the room required in the operating theatre and the little improvement over phacoemulsification are the main factors limiting the diffusion of the femtosecond laser [3].

The femtosecond laser acts by producing microbubbles inside the transparent tissues, and several microbubbles aligned in a ribbon produce a cut. The volume of the microbubbles depends on the laser energy and spacing, and one problem of the femtosecond laser use is to determine the precise amount of spot energy and spacing to obtain the cut. Lower energy than required will be ineffective, and excessive energy will result in large and confluent bubbles.

Since the spot energy must be set before surgery according to the supposed cataract hardness, it is possible to use an excess of energy with bubble formation that might damage the surrounding tissues. A specific problem of FLACS is how close to the posterior capsule (PC) fragmentation should be to fragment the cataractous lens while avoiding the rupture of the posterior capsule. After 10 years of FLACS, we identified some of the conditions that can place the posterior capsule at risk during femtosecond laser fragmentation and decided to share our experience and thoughts.

## Materials and methods

In our practice of femtosecond laser-assisted cataract surgery, for lens fragmentation the spot energy was set at 7 μ, the horizontal spacing at 5 μ and the vertical spacing at 4 μ. The posterior untreated safety zone was set at 0.7 mm. The laser we use (Victus, Bausch & Lomb, Germany) incorporates a swept-source OCT providing a lateral view of the laser’s surgical procedure. Lens fragmentation can be directly observed while progressing from the back to the front of the cataract. The OCT also gives an image of the posterior capsule, and any movement can be observed in the laser monitor. The surgeon has an immediate view of the cataract zone close to the posterior capsule and that of the posterior capsule dynamics allowing close observation during the surgical procedure.

The anatomical changes of the posterior lens with special attention to bubble formation were noted in relation to the supposed cataract hardness. The movements of the posterior capsule were evaluated and grouped trying to identify categories in relation to the laser energy and the characters of the cataract. In addition, we considered that the posterior capsule could be cut by the laser.

## Results

From July 12, 2012, to July 12, 2022, we performed 1465 FLACS procedures. In 1 eye, the posterior capsule was cut by the laser during lens fragmentation. The observation of the OCT videos providing a lateral view of the surgery allowed some understanding of the posterior capsule dynamics. We identified 4 different types of dynamics involving a rupture or the risk of rupture for the posterior capsule.

### Type 1: posterior capsule cut

**Fig. 1:**
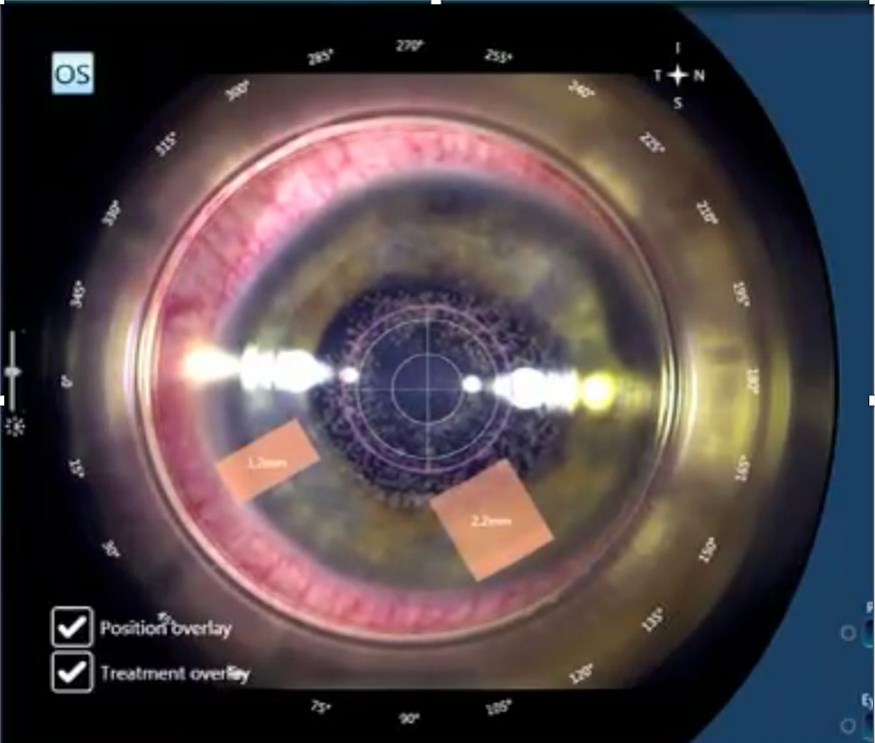
The eye became tilted after capsulotomy, when the fragmentation volume was already set. This resulted in posterior capsule partial cut by the femtosecond laser

A male patient aged 58 came for cataract surgery in the second eye after successful surgery in the first eye. Eye docking and capsulotomy were uneventful. After the capsulotomy, some movement of the eye was noted by the laser technician who suggested the surgeon to stop the procedure (Fig. 1). The surgeon decided to continue, but a partial cut was discovered in the inferior peripheral posterior capsule during surgery. The cataract was removed, and a 3-piece intraocular lens was implanted in the ciliary sulcus. Later on, a retinal detachment developed that required 2 further surgeries. As of the latest visit 2 years later, the retina was attached with 0.1 LogMAR corrected visual acuity.

### Type 2: posterior capsule movement

**Fig. 2:**
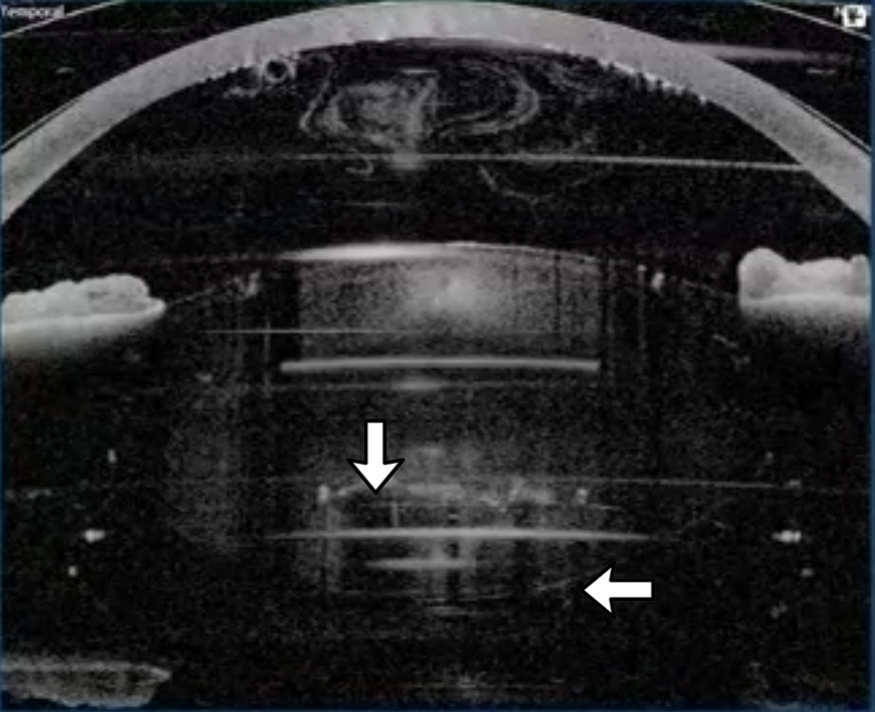
Some movement of the posterior capsule is normal during FLACS. (Arrows indicate the posterior capsule and the microbubbles produced by the femtosecond laser.) See also Video 2 in supplementary material

During our experience, we observed that some movement of the posterior capsule can be observed in most eyes at the beginning of the lens fragmentation. Posterior capsule movement was noted in 100% of the eyes with a hard cataract, and in lower percentages with lower cataract hardness. The suggested mechanism is the increase in volume induced by the gas microbubbles produced by the femtosecond laser (Fig. 2, Video 2). The size of these microbubbles depends on the spot energy and on the density of the lens tissue. Actually, the increase in lens volume during fragmentation is the reason to firstly perform the anterior capsulotomy. Noteworthy is the Ziemer femtosecond laser that uses minimal spot energy performs the lens fragmentation first [4].

### Type 3: posterior capsule concussion

**Fig. 3:**
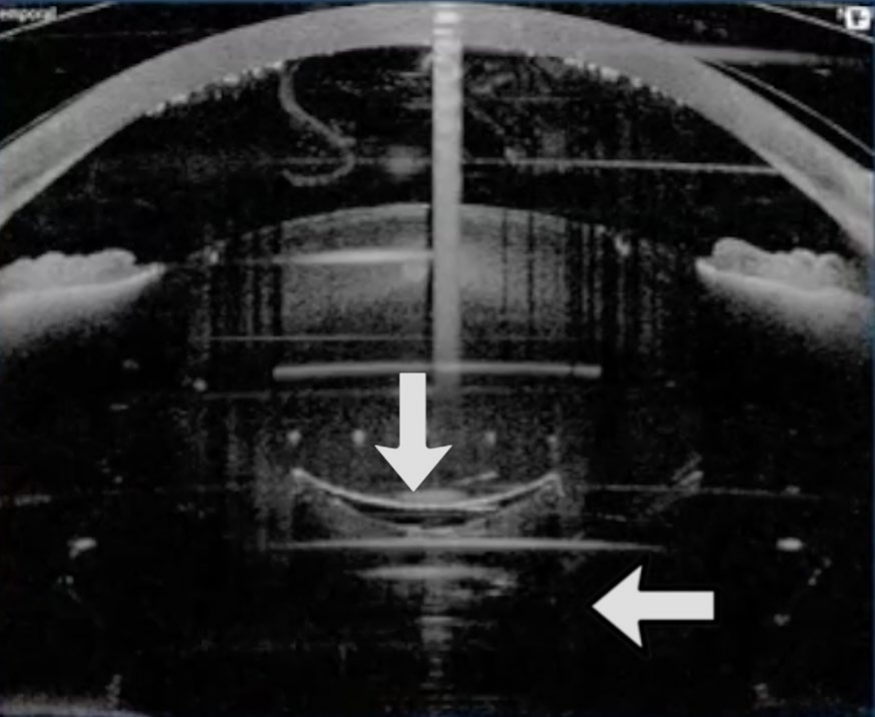
A large gas bubble resulted in concussion of the posterior lens capsule, with transient posterior displacement especially in the central part (white arrows). See also Video 3 in supplementary material

Instantaneous posterior displacement of the posterior capsule takes place when the cataract nucleus is very hard and a large gas bubble develops in the posterior lens cortex. The sudden increase in volume cannot displace the above standing lens and displaces posteriorly the posterior capsule. Spot energy higher than required and lens hardness favour this mechanism that appears to displace not a single part of the posterior capsule but all of it (Fig. 3, Video 3). Since the entire posterior capsule is displaced, the risk of a capsule rupture is relatively low. This formation was noted especially in hard cataracts in which the ability of the hard lens material to absorb part of the gas is minimal. In addition, the complex anterior hyaloid/vitreous offers lower resistance to gas expansion than a hard and large nucleus not yet fragmented, thus allowing posterior capsule displacement.

### Type 4: localized posterior capsule concussion

**Fig. 4:**
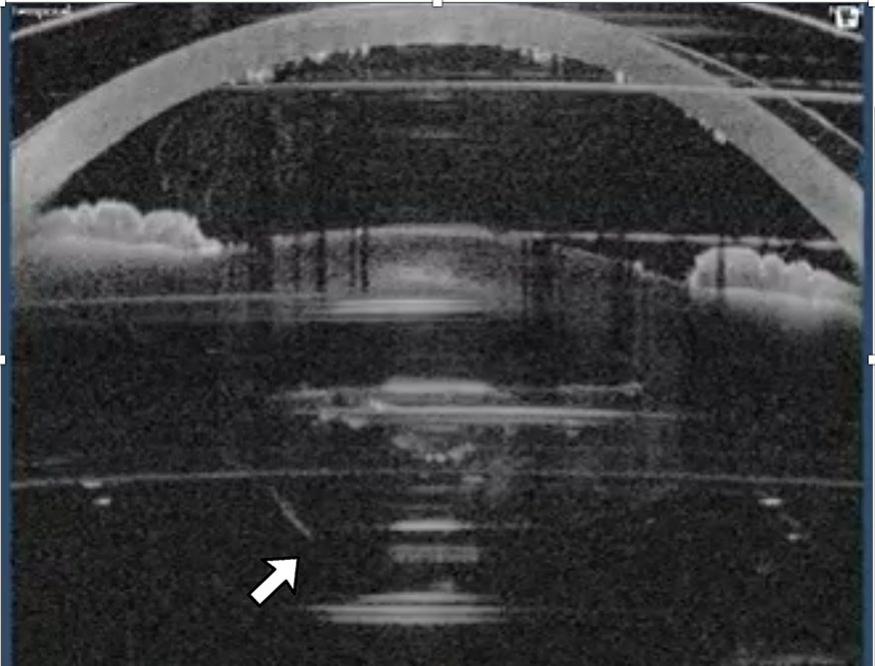
The posterior capsule had sudden posterior movement at the beginning of fragmentation also changing its profile in this case (white arrow). See also Video 4 in supplementary material

Localized displacement of the posterior capsule during concussion took place especially in association with the formation of large gas bubbles in the posterior part of the lens. This transient displacement is usually localized in the central portion of the posterior capsule, but it can take place also in the periphery if the pupil and the fragmentation area are large enough or if the lens material is nonhomogeneous (Fig. 4, Video 4). When fragmentation begins, even in part within the posterior cortex material the large gas bubble may expand peripherally and the risk for posterior capsule explosion may become real.

## Discussion

The behaviour of the posterior capsule during femtosecond laser lens fragmentation has received little attention so far. Most studies compared the number of posterior capsule tears between FLACS and phacoemulsification without further study of the dynamics of the tears [5].

The number of posterior capsule tears of FLACS is similar to that of phacoemulsification or even lower. In 2015, Abell et al. found no difference in the number of PC tears between FLACS and phacoemulsification [6]. Later on, Song et al. reported 0.65% unplanned vitrectomies in 2480 FLACS procedures, as compared with 0.62% in 36,865 phacoemulsification procedures [7]. More recently, Day et al. reported no PC tear in 391 FLACS eyes and 2 tears in 289 phacoemulsification eyes [8]. A recent survey confirmed the same complication rate for FLACS and for phacoemulsification [9].

Careful observation of the real-time OCT videos allows the assessment of the tissue dynamics during the whole femtosecond laser procedure, the capsulotomy, the lens fragmentation and the corneal incisions.

The only cut we had in the posterior capsule came from improper handling of a small eye movement after docking. Thorough control of 0° and 90° eye docking throughout the whole laser procedure is mandatory especially if we select a small posterior safety zone. It should be noted that values of 0.5 mm or lower are commonly adopted in FLACS.

The femtosecond laser action involves the formation of gas bubbles, thus increasing the volume of any treated tissue. The amount of this increase depends on spot energy, spacing and on tissue density. In FLACS, high energy produces larger bubbles; therefore, the minimal effective energy level is recommended. On the contrary, it seems reasonable to increase the spot energy to cut hard nuclei like we increase ultrasound power in phacoemulsification. However, the femtosecond laser energy is completely different from the ultrasound energy: it is light, stopped by opacity but very effective in hard transparent tissues that may require lower energy than softer opaque tissues. The large bubbles that displace posteriorly the posterior capsule for a few seconds develop from the excessive energy applied in the rear part of a hard lens nucleus. We can limit this displacement by reducing the spot energy, and by increasing the posterior safety zone in order to start fragmentation within the lens nucleus. Unfortunately this will also limit the efficacy of fragmentation.

We believe that the better understanding of the tissue dynamics during femtosecond laser activity is of importance not only in the setting of the surgeon’s preferred parameters, but also for the advancement of the interaction between engineers and clinicians. For instance, a setting of the spot energy increasing from the posterior lens cortex up to the equator zone and decreasing thereafter up to the anterior cortex might reduce the risk for the posterior capsule while maintaining the fragmentation efficiency.

## Supplementary Information

Below is the link to the electronic supplementary material.

Supplementary file1 (MP4 4182 KB)

Supplementary file2 (MP4 4734 KB)

Supplementary file3 (MP4 3819 KB)

## Data Availability

All data and material are available from the corresponding author.
